# Nutritional Risk Screening in Gynaecologic Oncology Surgery: Importance, Scoring Systems, Recommendations and Practical Applications

**DOI:** 10.3390/jcm15072622

**Published:** 2026-03-30

**Authors:** Laura R Caley, Iman Mustafa, Oliver Jagus, Helen Hutchinson, Amudha Thangavelu, Timothy Broadhead, David Nugent, Alexandros Laios

**Affiliations:** 1 Nutrition and Dietetic Department, St James University Hospital, Leeds LS9 7TF, UK; 2 Leeds Institute of Medical Research at St James’s, University of Leeds, Leeds LS9 7TF, UK; 3 Department of Gynaecological Cancer Surgery, St James University Hospital, Leeds LS9 7TF, UK; 4 Nutrition and Dietetics Service, University College London Hospitals NHS Foundation Trust, London NW1 2BU, UK

**Keywords:** gynaecologic oncology surgery, nutritional screening, malnutrition, perioperative nutrition, body composition

## Abstract

**Background/Objectives:** Malnutrition is common among women undergoing gynaecologic oncology (GO) surgery and is associated with increased morbidity, prolonged hospitalisation, and reduced survival. Nevertheless, the optimal nutritional screening tools remain uncertain. **Methods:** We conducted a narrative review of commonly used nutritional screening and assessment tools in surgical GO patients. To highlight practical challenges in accurately identifying at risk individuals, we incorporated findings from our clinical audit. **Results:** There was a considerable variation between tools. While many tools were associated with adverse outcomes, their clinical value in this population was unclear. The presence of ascites and rapid deterioration in oral intake may contribute to under-recognition of at-risk patients, as illustrated by our audit findings. Emerging strategies including determining body composition from routine pre-operative Computed Tomography (CT) scans, which have shown statistical associations with survival and toxicity in observational studies, but their clinical utility is not yet established. **Conclusions:** Although several screening tools were statistically associated with adverse outcomes, robust data on their clinical utility are lacking. Current tools may inadequately factor for the specific considerations when screening this group. Consequently, nutritionally vulnerable surgical GO patients requiring nutritional intervention may be missed. As no gold standard currently exists for this population, bespoke, objective approaches and prospective studies are urgently needed to address disease-specific nutritional considerations.

## 1. Introduction

Malnutrition, herein referring to protein–energy undernutrition, can be defined as deficit nutrient balance to the extent that it causes measurable ill-effects on body composition, functionality, and/or clinical outcomes [1]. Malnutrition is common in cancer patients owing to metabolic and inflammatory shifts, reduced oral intake, and tumour-site dependent, impaired assimilation of nutrients. This can be exacerbated by cancer treatments, including surgery, further elevating nutritional requirements and deteriorating intake. The deleterious impacts of malnutrition may be attributable to the reduced immune and metabolic reserve required to counteract the significant catabolic stress of surgery, potentially compounded by the severity of malnutrition being indicative of a more advanced disease state. This is particularly important as severe malnutrition has been independently associated with increased mortality in cancer patients [2].

In women undergoing surgery for a gynaecological oncology (GO) cancer, malnutrition is particularly prevalent. Malnutrition is associated with poorer surgical outcomes, including increased perioperative morbidity and delayed recovery [3,4,5]. Significant nutritional challenges can arise in this patient group owing to the direct impact of tumour burden on oral intake, including low appetite, early satiety, and gastrointestinal symptoms. This is further exacerbated by disease- and treatment-related metabolic demands and side effects. Prompt identification and management of nutritional risk is paramount, including consideration of delaying surgery for pre-operative nutritional therapy in cases of severe malnutrition [6]. Nutritional intervention has the potential to decrease post-operative complications, but randomised control trials on nutritional interventions in this patient group are lacking [7,8] Guidelines recommend regular nutritional screening pre- and post-major surgery [6].

Given the complexity of malnutrition, a plethora of nutritional screening tools exist. Their aims can vary, from identifying individuals at risk of protein–energy malnutrition requiring nutritional input, to prognostic indices and risk stratification. Some nutritional screening tools also embed elements of nutritional assessment (e.g., investigation of aetiology). These distinctions are important to appreciate for appropriate selection for use. Within the clinical setting, tools are primarily employed to identify patients at risk of protein–energy malnutrition to trigger prompt nutritional intervention. The choice of nutritional screening approach is usually shaped by local policies, and the need for quick and practical bedside assessment. They typically include measures of body mass index, percentage of unintentional weight loss, and impairments in oral intake.

However, accurately screening GO surgical patients presents challenges, including the impact of ascites on body weight and oral intake. This can be further exacerbated by rapid peri-operative nutritional changes, owing to elevated nutritional requirements, often coupled with reduced appetite, catabolic stress from extensive surgery, gastrointestinal symptoms, and potential post-operative ileus. These factors can influence the accuracy of standard nutritional screening approaches and obscure the need for timely specialist nutritional input. It remains unclear which nutritional screening tools best guide clinical decision-making, defined as their utility in reliably identifying patients who would benefit from nutritional intervention. Similarly, various approaches exist to predict clinical outcomes, but their clinically utility has not been thoroughly addressed.

To further explore this topic, we aim to review the current evidence on nutritional screening and assessment tools in surgical GO patients, highlighting both statistical associations and current limitations in clinical applicability. We incorporate findings from our recent clinical audit evaluating the accuracy of nutritional screening in this patient group to inform future recommendations.

## 2. Materials and Methods

### 2.1. Literature Search

A literature search was conducted across the following databases: PubMed, Web of Science, and MEDLINE on 23 March 2025. Full search terms for each database are presented in Appendix A Table A1. Full eligibility criteria are presented in Appendix A Table A2. In brief, studies on nutritional assessment or screening in GO surgical patients in the pre-, peri-, or post-operative period were included. A narrative review was undertaken, with studies too heterogeneous in nature to undertake meta-analysis.

### 2.2. Clinical Audit

A retrospective audit was undertaken to evaluate the accuracy of routine nutritional screening scores compared to formal dietetic assessments in GO surgical patients. The audit was conducted in a United Kingdom (UK) tertiary specialist centre, where nursing staff performed nutritional screening based on the Malnutrition Universal Screening Tool (MUST) upon admission and weekly thereafter [9]. The MUST score recorded closest to the time of dietetic referral was compared to the GO surgical dietitian’s initial assessment (patients under another dietetic team during admission were excluded, n = 6). The data, collected from November 2023 to April 2024, was registered in the Local Audit Database (LOC0822).

## 3. Results

### 3.1. Literature Review

**Figure A1:**
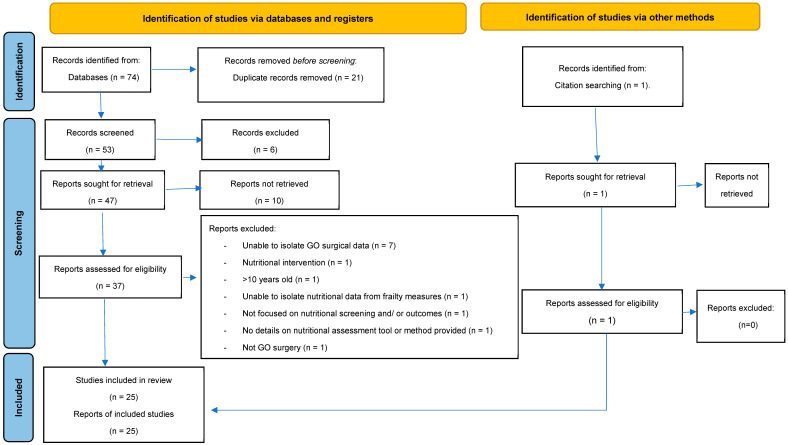
PRISMA 2020 flow diagram [47].

After removing duplicates, 53 records were screened, of which 25 met the inclusion criteria (Appendix A Figure A1). A summary of key characteristics for each study is presented in Table 1. The most commonly employed nutritional screening and/or assessment methods were: Nutritional Risk Index-2002 [10,11,12,13]; Malnutrition Screening Tool [14]; Academy of Nutrition and Dietetics—American Society for Parenteral and Enteral Nutrition [14]; Subjective Global Assessment (SGA) [15]; Global Leadership Initiative on Malnutrition (GLIM) [15]; anthropometrics [12,15,16,17,18,19,20]; body composition [12,15,21,22,23,24]; patient generated-SGA [13,16,17,21,22]; Malnutrition Universal Screening Tool [18] and biochemical indicators [12,13,16,17,18,20,23,25,26,27,28,29,30,31,32,33,34]. These tools will be discussed within the context of clinical expertise.

#### 3.1.1. Anthropometrics and Body Composition (BC)

Physical measurements were undertaken by several studies, often in tandem with other approaches [12,15,16,17,18,19,20]. Two studies defined a body mass index (BMI) < 18.5 kg/m^2^ as malnourished [12,20], while another study employed a cut-off < 22 kg/m^2^ [17]. Debate exists regarding the most appropriate BMI cut-off values, particularly for certain ethnicities, age groups and disease states, with not widely accepted GO surgical-specific recommendations [35,36]. Similarly, two studies defined a >5% weight loss in the last six months as malnourished [18,19]; however, many other widely accepted screening tools would not count <10% weight loss in isolation as a high risk of malnutrition [9]. Another study calculated percentage weight loss within a one-month period [16]. This underscores the difficulty of relying on a single anthropometric measure to assess nutritional risk. For this reason, additive factors are used in most nutritional screening approaches, as shortly explored. Imperatively, a significant proportion of women with GO cancers experience ascites, which hampers accurate determination of BMI and unintentional weight loss, rendering singular measures likely insufficient.

Body composition measures offer a more accurate insight into body proportions. This was explored in a number of studies [12,21,22,23,24], some of which also included anthropometrics [15,16]. One study calculated fat mass index (FMI) and fat-free mass index (FFMI) from waist and hip circumference and skinfold thickness pre- and post-GO surgery, showing significant reduction in these parameters, along with BMI [15]. Handgrip strength was measured during hospital admission in one cross-sectional study, which found it predicted malnutrition [16]. Another study measured BC with bioelectrical impedance analysis (BIA) pre-operatively in 70 women with advanced epithelial ovarian cancer, showing significantly lower values in those with residual disease and peri-operative complications [24]. Nevertheless, considering the high volume of ascites in this patient group, the BIA approach may not be the most accurate means of assessing BC. As an alternative, BC can be directly measured from routine clinical Computed Tomography (CT) scans. Two retrospective studies found that BMI and skeletal muscle index (SMI) were significantly decreased post-surgery, and were associated with higher radiotherapy toxicities [21,22]. A case note review of ovarian cancer patients treated with debulking surgery and adjuvant chemotherapy found that SMI loss was independently associated with all-cause mortality [23].

#### 3.1.2. Malnutrition Screening Tool (MST)

The MST records self-reported unintentional weight loss (in raw values), and binary response regarding reduced oral intake due to decreased appetite. While simple, it may be less sensitive than MUST [37]. One study found that only 15% of GO patients who screened positive on the MST in the outpatient setting were identified as ‘at risk’ on a hospital screening tool [14]. Importantly, in this patient group, fluid shifts may mask dry weight loss.

#### 3.1.3. Nutritional Risk Screening (NRS)-2002

The NRS-2002 consists of initial screening and, if indicated, final screening. Initial screening assesses BMI, weight loss in the last three months, reduced oral intake in last week, and disease severity. It is endorsed by the European Society of Clinical Nutrition and Metabolism (ESPEN) [38]. In pre-operative hospital screening of GO patients, those identified as malnourished on NRS-2002 had a longer hospital stay [10]. Limitations of NRS-2002 include inconsistent validity in different hospital populations and age groups [37]. The prevalence of pre-operative malnutrition varied between 19–70% according to NRS-2002 in papers identified in this review [11,12]. This variability may be attributed to several clinical factors. For instance, Nasser et al. [12] observed that patients aged >65 years old, with ascites exceeding 500 ml and platinum-resistant disease, had a significantly higher risk of elevated NRS-2002 scores. These higher scores were, in turn, associated with reduced progression-free and overall survival [12]. Such clinical heterogeneity complicates the interpretation of nutritional risk across the GO surgical population.

#### 3.1.4. Subjective Global Assessment (SGA)

Nutritional parameters in SGA include percentage weight loss in the last two weeks and six months, degree of change in nutrient intake, presence of symptoms affecting oral intake, functional capacity, metabolic demand alongside a physical examination for indices of muscle wasting, loss of subcutaneous fat, and the presence of oedema or ascites. One study that employed the SGA found that 17% of GO surgical patients were mildly or moderately malnourished preoperatively, rising to 34% post-operatively [15]. There were also significant reductions in BMI, waist and hip circumference, triceps thickness, body fat, fat mass, and FMI post-surgery, suggesting that the assessment tool detects nutritional status changes [15]. Notably, the study also assessed nutritional status using the GLIM criteria and found that malnutrition prevalence in the same patient group increased from 7.5% to 15.1% pre- and postoperatively, respectively [15]. This highlights the substantial prevalence of malnutrition amongst GO patients, which is further accentuated by surgery. However, significant variability existed between screening and assessment tools. The SGA was not ubiquitously used in the hospital setting, potentially owing to its complexity.

#### 3.1.5. Patient-Generated Subjective Global Assessment (PG-SGA)

The PG-SGA is completed by the patient. While several studies employed the PG-SGA, malnutrition detected varied between 10–57% [16,22]. The observed differences were likely influenced by varying clinical characteristics and the timing of nutritional assessment. This is because the PG-SGA assesses dietary changes over the preceding 2–4 weeks, which may inadequately reflect the rapid nutritional deterioration post-surgery. Additionally, questions pertaining to activity levels may be less applicable following major surgery, when patients are typically immobilised or acutely unwell and therefore this tool might not be ubiquitously employed across the GO surgical group. This is an important consideration as onward referrals often depend on nutritional screening scores. Inconsistencies between tools may yield differing responses, potentially impacting nutritional care.

#### 3.1.6. Albumin and Other Biochemical Markers of Nutritional Status

Biochemical markers were commonly employed by studies and were linked to survival outcomes [20,25,26,27,28,29,30,31,32,33]. However, attributing their findings to nutritional status is challenging, particularly given the retrospective nature of these studies and the confounding effect of the disease burden. One study found that hypoalbuminemia (<35 g/L) did not correlate with percentage weight loss, NRI or MUST nor intra- or post-operative complications or 30-day mortality [18]. Similarly, preoperative albumin was not significantly associated with the PG-SGA score in 97 GO patients planned for surgery [17]. Another study found that 27% were malnourished according to albumin levels (≤30 g/L) compared to 47% using the PG-SGA [13], with the discordance suggestive of distinct measures.

Clinically, albumin and other biochemical markers are rarely considered direct markers of nutritional status. Albumin is an acute-phase reactant, and its production is downregulated with the metabolic stress of surgery. Further hampering the accuracy of albumin in this patient group is the dilutional effect of ascites and oedema on serum levels. Similarly, other blood markers, such as white cell count and haemoglobin, are too intrinsically linked with the surgery and the underlying disease itself to be considered independent markers of nutritional status. Therefore, biochemical values must be considered within the clinical context. For example, a systematic review of severely undernourished, but otherwise healthy individuals, found serum albumin levels were normal [39].

#### 3.1.7. Academy of Nutrition and Dietetics—American Society for Parenteral and Enteral Nutrition (AND-ASPEN) Criteria for Malnutrition and Global Leadership Initiative on Malnutrition (GLIM)

The AND-ASPEN criteria for malnutrition include inadequate energy intake, weight loss, muscle and subcutaneous fat loss, fluid accumulation (including ascites), and reduced functional status [40]. While including many pertinent measures, it requires nutritional expertise for calculations and would be challenging to ubiquitously implement within a clinical setting without a dedicated dietitian. One study employed AND-ASPEN criteria for malnutrition, measured by a dietitian, in conjunction with an in-house hospital nutritional screening tool [14]. Despite its comprehensive design, only 4% out of 107 ovarian cancer patients undergoing surgical cytoreduction were identified as malnourished according to AND-ASPEN, compared to 15% with an in-house hospital screening tool [14].

The GLIM recommends a two-step model; the first involves screening and those ‘at risk’ with any validated screening tool, the second employing their diagnostic assessment criteria and severity grading approach [41]. The latter diagnostic assessment includes weight and muscle mass loss, low BMI, and reduced food intake along with disease burden [41]. Akin to AND-ASPEN, aetiological criteria would necessitate specialist nutritional knowledge, impeding its more global use within the hospital setting. One study employed GLIM criteria and reported a 7.5% and 15.1% prevalence of malnutrition pre- and post-operatively, respectively, which was approximately half that identified by SGA in this cohort [15].

#### 3.1.8. Malnutrition Universal Screening Tool (MUST)

MUST is commonly used, and is endorsed by ESPEN [36]. It assesses BMI, percentage of unintentional body weight loss, and actual or anticipated minimal nutritional intake for ≥5 days [9]. Frequently, only those identified as ‘high risk’ for malnutrition meet dietetic referral criteria [9]. The strengths of MUST include its simplicity, strong validation, and outperformance of other similar tools [37,42,43].

One retrospective study found no correlation between MUST score and post-operative morbidity [18]. However, the high volume of ascites in these surgical GO patients hampers accurate assessment of both BMI and percentage weight loss. Although mid-upper arm (MAC) could be considered as an alternative, interpreting the degree of undernutrition by this measure remains challenging [38]. From clinical experience, MAC is infrequently employed in hospital wards and patient groups where weight can be obtained, albeit inaccurately. There is also no section in the MUST tool detailing barriers to oral intake outside of minimal intake. Based on clinical experience, the tool lacks specificity for this patient group, prompting further investigation via a local audit.

### 3.2. Clinical Experience: An Audit of Accuracy of Nutritional Screening GO Surgical Patients in a UK Tertiary Hospital

We carried out a retrospective audit assessing the accuracy of nutritional screening scores in surgical GO patients compared to subsequent dietetic assessments. Of the 34 dietetic referrals analysed, all women with a GO diagnosis had undergone elective midline laparotomies. Referrals were made on average five days post-admission (SD 3.5). A key finding was that most dietetic referrals were prompted by the wider multidisciplinary team (MDT), based on clinical judgement, rather than by the nutritional screening itself. Nutritional screening was completed in 28 (82%) patients (national target 100%); however, only two (7%) were identified as high risk by nursing staff. A re-calculation based on dietetic records identified 24 patients (71%) as high risk. Acute deterioration in oral intake post-surgery was the main driver of high-risk classification, present in 83% of these patients (20/24). For eight patients (33%), ascites interfered with weight assessments. These discrepancies were not adequately captured by initial screening.

The limitations of the audit included small sample size, retrospective design, and lack of contemporaneity between screening and dietitian assessments. Improved harmonisation of nutritional screening approaches would enable more accurate data collection on nutritional parameters within and between countries to identify areas for enhancing surgical outcomes. The audit rationale assumes that improved screening will directly lead to better outcomes, but the projected impact needs be explored. Nevertheless, our audit revealed that nutritional screening scores were not an accurate reflection of nutritional status at the time of dietetic assessment, mainly due to acute declines in oral intake, not detected by weekly screening. Recommendations for clinical practice are detailed in Table 2.

## 4. Discussion

Malnutrition is both common and costly amongst women with gynaecological cancer. The degree of severity of malnutrition has been statistically associated with poorer surgical outcomes [3,4]. While several nutritional screening and assessment tools are widely used in practice, whether they are optimal to detect malnutrition in GO surgical patients remains unknown.

Accurate and timely nutritional screening and assessment are statistically associated with reduced complications, shorter length of stay and improved treatment tolerance [38]. Nutritional screening has the potential to be clinically meaningful because it identifies patients who may benefit from prehabilitation, dietetic input and peri-operative nutritional support. In GO surgery, where procedures are often prolonged and demanding, failure to identify malnutrition can delay intervention resulting in increased risks of refeeding syndrome, ileus and prolonged recovery. High-quality randomised trials in this exact population are limited. This is framed as a research gap rather than a weakness of the principle of screening per se, and highlights the need for an appropriate, evidence-based approach for this patient group. Practice is often dictated by local protocols, national guidelines, and practitioner familiarity. A gold standard is lacking and no widely accepted tool currently exists, that is, one that reliably identifies nutritionally vulnerable patients and informs tailored nutritional management across the surgical pathway.

In GO surgery, a fundamental consideration is ascites, which can mask true bodyweight and hinder accurate calculation of BMI and percentage of dry body weight loss, thereby limiting sensitivity and specificity. Our local audit underscores this gap, whereby initial nutritional screening with MUST was not reflective of nutritional status at the time of dietetic assessment. This discrepancy suggests that standardised tools may not adequately and dynamically capture malnutrition in this population; this is especially the case in the early post-operative phase where acute deteriorations may arise. Routine CT-based BC analysis has been explored in case note reviews as a more objective method for assessing nutritional status. Several studies showed statistical associations with BMI, toxicities, and all-cause mortality [21,22,23]. Specific to ovarian cancer patients, CT-based BC measures have been correlated with survival rates [4], hospital length [44], and chemotherapy complications [45]. While these associations are encouraging, CT-based assessments are predominantly confined to the research setting. Their clinical utility to inform decision-making or improve outcomes has yet to be prospectively demonstrated. Advances in artificial intelligence-based automation could provide sophisticated assessments of BC and refine nutritional screening [46]. However, practical considerations such as software availability, clinician training, and resource implications must be addressed before routine implementation.

An additional consideration is the integration of BC measures with tailored novel nutritional screening tools specifically designed for the surgical GO group, particularly in the peri-operative period, when BC measures would not be routinely or easily obtained within the acute hospital setting. Disease-specific nutritional screening tools are available for liver and renal disease. In this context, our audit findings suggest that more frequent re-screening may be necessary to detect rapid postoperative nutritional changes.

This literature review highlights limitations in interpreting current research in the area. This includes discordant timing of screening and assessment during the GO surgical pathway, different disease stages, and anticipated variations in practice and outcomes between countries and institutes, potentially influencing findings. Further complicating assessment of these tools is their varying and potentially overlapping aims, e.g., disease stratification versus identifying individual patients where nutritional input is indicated and/or may influence clinical outcomes. Therefore, the results should be interpreted with due caution, and these findings highlight the need for more harmonised, standardised approaches to enhance evidence-based clinical practice in this area. Limitations of the narrative review also include the inability to formally assess study quality and risk of bias, owing to the heterogeneity of the included studies. The limitations of the clinical audit include retrospective design, small sample size, and the difference in timing between nursing and dietitian assessment. Dietitian assessments are also not infallible, although they are experts in nutritional assessment. The audit results should be viewed as preliminary and hypothesis-generating.

In conclusion, while nutritional assessment is a critical component of peri-operative care, its present form may not adequately identify all those at high risk of severe malnutrition amongst surgical GO patients. Future research should focus on validating screening tools for clinical utility and explore the integration of objective measures such as CT-derived BC to improve nutritional care and surgical outcomes.

## Figures and Tables

**Table 1:** Characteristics of included studies.

	Reference	Design	Country	Nutritional Screening/Assessment Tools	Patient Group	Key Findings
1	Aynaci and Guksu (2019) [10]	Prospective cohort study	Turkey	NRS-2002 (score > 3 indicating severe malnutrition) Completed within 48 h of surgical hospital admission	N = 334 gynae-oncology surgical patients	29% prevalence of severe malnutrition preoperatively Prolonged hospital length in those with malnutrition compared to those not identified at risk
2	Benoit et al. (2024) [18]	Retrospective case note review	France	Weight loss in the last six months (>5%), albuminemia (<35 g/L), NRI ^1^ (score 97.5–100 defined as mild malnutrition, 83.5–97.4—moderate malnutrition, <83.5 indicating severe malnutrition) and MUST (Score 1 indicating medium risk and ≥2 high risk for malnutrition) all measured at time of diagnosis	N = 615 epithelial ovarian cancer of whom n = 533 underwent surgery	No correlation between % weight loss, albuminemia, NRI or MUST and: intra- or post-operative complication or 30-day mortality
3	Chantragawee and Achariyapota (2016) [17]	Cross-sectional study	Thailand	PG-SGA (score 4–8 defined as moderately or suspected of being malnourished and score >8 defined as severely malnourished) and association with BMI (low defined as <22 kg/m^2^) and serum albumin (with <4 g/dL defined as low) Measured pre-operatively at pre-anaesthesia assessment	N = 97 gynaecological cancer patients planned for surgery	PG-SGA identified 54% malnourished, of which 21% severely malnourished Pre-operative BMI and albumin were not significantly associated with PG-SGA score
4	Chen et al. (2025) [25]	Retrospective case note review	China	PNI ^2^ and coPNI-SII measured within one week before surgery	N = 154 epithelial ovarian cancer who underwent fully staged surgery	PNI and coPNI-SII predictor of survival after surgery
5	Eurich et al. (2022) [14]	Quality improvement project	United States	Outpatient setting: MST. If positive (answered yes to either question), offered outpatient dietitian assessment employing AND-ASPEN criteria of malnutrition (severe malnutrition defined as score of ≥2 in severe category, ≥2 in moderation category or 1 severe and 1 moderate defined as moderate malnutrition) Inpatient setting: Dietitian assessment employing AND-ASPEN criteria immediately after surgery and nursing administered hospital nutritional screening tool (in-house tool)	N = 107 ovarian cancer undergoing surgery	Inpatient setting 14% screened positive for malnutrition on hospital screening tool 4% identified as malnourished according to AND-ASPEN criteria 15% of patients with positive MST as outpatient identified as ‘at risk’ on hospital screening tool
6	Feng et al. (2018) [26]	Retrospective case note review	China	PNI ^2^ measured within one week before surgery	N = 875 undergoing primary staging or debulking surgery for high grade serous ovarian cancer	Low pre-operative PNI associated with increased overall survival
7	Fumagalli et al. (2024) [27]	Retrospective cohort study	United States	Albumin and PN2 ^1^ measured within 45 days prior to surgery	N = 675 ovarian cancer patients undergoing primary cytoreductive surgery	Albumin < 3.5 g/dL and PNI < 45 each associated with 90-day morality
8	Gounitsioti et al. (2022) [15]	Prospective cohort study	Greece	SGA (category B mild or moderately malnourished and category C indicated severe malnutrition) and GLIM ^4^, BMI status, waist and hip circumference, skinfold thickness for calculation of FMI and FFMI measured pre- and post-surgery	N = 53 gynae-oncology surgical patients	Significant reductions in body weight, BMI, waist and hip circumference, triceps thickness, body fat, fat mass, fat mass index post-surgery Significant increase in malnutrition status for both SGA and GLIM score post-surgery SGA: 17% mildly or moderately malnourished preoperatively and 34% post operatively GLIM: 7.5% positive for malnutrition pre-operatively and 15.1% post-operatively
9	Hertlein et al. 2014 [11]	Prospective cross-sectional study	Germany	NRS-2002 on day of hospital admission (score ≥ 3 indicates severe impaired nutrition status)	N = 106 gynae-oncology surgical patients	NRS ≥ 3 present in: - 70% ovarian cancer - 60% endometrial cancer - 32% cervical cancer - 33% vulva cancer
10	Ho et al. (2021) [16]	Cross-sectional study	Malaysia	Anthropometrics (BMI, % weight loss in 1 month, FM, FFM, muscle mass, MUAC), biochemistry (CRP, album), total daily energy and protein intake, handgrip strength, PG-SGA measured during surgical hospital admission (for PG-SGA: malnourished defined by either SGA B or SGA C)	N = 124 gynaecologic cancer surgical patients	57% malnourished according to PG-SGA % weight loss in last month, and handgrip strength both significant predictions of malnutrition (along with haemoglobin and CRP)
11	Kim et al. (2023) [28]	Retrospective case note review	Korea	PNI ^2^ measured within 1 month pre-operatively	N = 894 Endometrial cancer surgical patients	High pre-operative PNI associated with post-operative cancer specific survival
12	Lee et al. (2021) [21]	Retrospective case note review	Taiwan	PG-SGA (divided into two groups for analysis: score 0–3 well-nourished and ≥4—combination of categories B and C—as ‘at risk of malnutrition’) Skeletal muscle index from CT scans at L3 vertebral level pre-surgery and pre- and within 3 months post-radiotherapy	N = 210 patients undergoing surgery for cervical or endometrial cancer and then post-operative pelvic radiotherapy +/− chemotherapy	Significant decrease in BMI, SMI and TATI post-surgery Higher PRO-CTCAE associated with significantly reduced BMI and SMI after surgery
13	Lee et al. (2022) [22]	Retrospective case note review	Taiwan	PG-SGA (divided into two groups for analysis: score 0–3 well-nourished and ≥4—combination of categories B and C—as ‘malnourished’) Skeletal muscle index from CT scans at L3 vertebral level pre-surgery and pre- and within 3 months post-radiotherapy	N = 133 early-stage cervical cancer undergoing surgery and then adjuvant radiotherapy +/− chemotherapy	Significant decrease in BMI, SMI post-surgery 10% malnourished according to PG-SGA post-surgery Higher PRO-CTCAE associated with significantly reduced BMI and SMI after surgery
14	Lee et al. (2023) [23]	Retrospective case note review	Taiwan	PNI ^2^ measured within 1 week before surgery SMI measured using CT scans at L3 vertebral level pre- and post- treatment. CT scans were within 2 weeks pre-surgery and within 3 months after adjuvant chemotherapy	N = 650 ovarian cancer patients treated with primary debulking surgery and adjuvant chemotherapy	Low PNI and SMI loss were independently associated with all-cause mortality
15	Li et al. (2021) [29]	Retrospective cohort study	China	NPS ^3^ measured within 15 days before surgery	N = 1038 patients with endometrial cancer undergoing surgery	NPS independent prognostic factors for progression-free survival and overall survival
16	Liu et al. (2025) [30]	Retrospective cohort study	China	PNI ^2^ measured within 7 days before surgery	N = 922 patients with epithelial ovarian cancer who received comprehensive staged or debulking surgery	High PNI was an independent protective factor for progression-free survival for early-stage patients PNI was an independent prognostic factor for overall surgical in advanced disease
17	Miao et al. (2016) [33]	Retrospective cohort study	China	PNI ^2^ measured pre-operatively	N = 344 patients with epithelial ovarian cancer who received comprehensive staged or debulking surgery and then post-operative adjuvant chemotherapy	PNI levels significantly different in those who responded to platinum-based chemotherapy (platinum-sensitive group—P-S) compared to those who did not (platinum resistant—PR), with mean being higher in the P-S group Those with a lower PNI (<45) had significantly lower progression free survival and overall survival PNI level significantly associated with BMI
18	Nasser et al. (2024) [12]	Prospective observational study	Germany	NRI ^1^ (score < 100 defined as malnourished), albumin (levels ≤ 4 g/dL defined as malnourished) and pre-albumin (with levels < 20 mg/L defined as malnourished) all measured on day 1 of admission BMI (with <18.5 kg/m^2^ defined as underweight), NRS-2002 (with score of ≥3 defined as high-risk for malnutrition) and bio-electrical impedance analysis to measure body composition—all measured pre-operatively	N = 152 patients with EOC cancer, tubal or peritoneal cancer admitted for primary, secondary, or tertiary cytoreductive surgery	18% identified as malnourished on NRS-2002, 19% experienced >5% weight loss in last 3 months, 32% and 35% NRI and albumin levels suggestive of malnutrition by cut of criteria of <100 and ≤4 respectively Significantly increased risk of malnutrition measured on NRS-2002: with ascites >500 mls, platinum-resistance and age >65 years old Lower progression free and overall survival with NRS-score ≥ 3
19	Ogura et al. (2022) [31]	Retrospective case note review	Japan	GNRI ^4^ measured immediately pre-operatively	N = 75 EOC patients undergoing surgery	Lower GNRI score associated with lower survival rate
20	Pache et al. (2019) [19]	Retrospective case note review	Switzerland	Pre-operative unintentional weight loss > 5% in the 6 months prior to surgery—either patient reported or recorded by primary care physician within two weeks of surgery	N = 339 undergoing major elective gynaecological surgery within the enhanced recovery after surgery (ERAS) programme in gynaecological oncology unit	Pre-operatively, 10% had a weight loss (>5%) loss ≥5% weight loss independent risk factor for postoperative complications in those undergoing more extensive surgical procedures and longer length of hospital stay
21	Pergialiotis, et al. (2024) [32]	Prospective cohort study	Greece	PNI ^2^ measured on admission to clinic and at least 24 h before surgery	N = 208 undergoing surgery for gynaecological cancer (ovarian, endometrial, cervical and vulvar)	PNI predicted risk of peri-operative infection
22	Tian et al. (2021) [13]	Prospective observational study	China	- NRS-2002 (with score ≥ 3 indicates a nutritional risk) - PG-SGA (score ≥ 4 defined as malnourished in study) - Both compared against serum albumin level (with ≤30 g/L defined as malnourished) - All measured on admission	N = 165 cervical cancer patients undergoing surgery	27% nutritionally at risk on NRS-2002 47% nutritionally at risk of PG-SGA 27% malnourished according to albumin levels
23	Uccella et al. (2018) [24]	Prospective observational study	Italy	Measurement of body composition with Bioelectrical impedance analysis (BIA) with BIA-derived phase angle (PhA) at 50 KHz on admission to hospital prior to surgery	N = 70 patients with newly diagnosed advanced (stage IIIC-IV) EOC	Pre-operative PhA was significantly lower in patients with residual disease and peri-operative complications
24	Zhang, et al. (2021) [20]	Retrospective case note review	China	CONUT ^5^ score collected from blood sample within 1 week before surgery BMI (cut off < 18.5 kg/m^2^ defined as underweight) PNI ^2^ (high score defined as >48.55)	N = 698 early-stage cervical cancer patients undergoing post-operative concurrent chemoradiotherapy	58% BMI < 18.5 kg/m^2^ Higher CONUT group independent predictor of disease free and overall survival Lower BMI and PNI in those with higher COUNT score
25	Zhu et al. (2024) [34]	Retrospective cross-sectional study	China	Serum albumin (low defined at <40 g/L) and PNI ^2^ (low score defined as <45) measured prior to surgical treatment	N = 155 GO patients who developed post-surgical lower limb lymphedema (LLL)	40% low serum albumin 81% low PNI scores

AND-ASPEN—Academy of Nutrition and Dietetics—American Society for Parenteral and Enteral Nutrition; BMI—body mass index; CONUT—controlling nutritional status; coPNI-SII—combination Prognostic Nutritional Index and Systemic Immunoinflammatory Index; CT—computed tomography; EOC—epithelial ovarian cancer; FM—fat mass; FFM—fat mass index, FFMI—fat-free mass index; GLIM—Global Leadership Initiative on Malnutrition; GNRI—geriatric nutritional risk index; MST—Malnutrition Screening Tool; MUAC—mid upper arm circumference; MUST—Malnutrition Universal Screening Tool; NPS—Naples prognostic score; NRI—Nutritional Risk Index; NRS-2002—Nutritional Risk Screening-2002, PG-SGA—patient generated subjective global assessment; PLR—platelet-to-lymphocyte ratio; PNI—Prognostic Nutritional Index; PRO-CTCAE—PRO version of the Common Terminology Criteria for Adverse Event; NLR—neutrophil-to-lymphocyte ratio; SGA—Subjective Global Assessment; SII—Systemic Immunoinflammatory Index; in patients, SMI—skeletal muscle index; TATI—total adipose tissue index. ^1^ NRI: biochemical scoring system to reflect nutrition, albumin, and present and usual weight. ^2^ PNI: biochemical scoring system to reflect nutrition and immune status, including serum albumin and peripheral blood lymphocyte count. ^3^ NPS: biochemical scoring system to reflect nutrition and immune status, including serum albumin, total cholesterol, neutrophil–lymphocyte ratio and lymphocyte–monocyte ratio. ^4^ GNRI: calculated from serum albumin level and percentage ideal body weight. ^5^ CONUT: biochemical scoring system to reflect nutritional status, including serum albumin levels, total cholesterol levels and total peripheral lymphocyte counts.

**Table 2:** Audit Recommendations for Nutritional Screening in Gynaecological Surgical Patients.

Recommendation	Rationale
Exploring barriers when nutritional screening	− There are many competing pressures for nursing staff; understanding their perspective and specific challenges encountered would support a more targeted, collaborative approach to improve screening
Develop methods to enhance weight history recording on the nutritional screening tool	− Weight history was infrequently recorded in detail on nutritional screening by nursing staff. Identifying barriers and providing training is indicated − Alongside this, more broad approaches should be considered, such as clinical records automatically pulling data on weight history − Weight history could also be more consistently collected pre-surgery at pre-assessment visit
Develop processes so that ascites is more routinely considered on nutritional screening	− Nursing team could enquire directly with GO surgical patients and liaise the surgical team regarding the present of ascites and consider alternative measures such as mid upper arm circumference − Volume of ascites recorded surgical notes could be utilised
Nutritional screening training for staff nurses and wider MDT	− The vast majority of referrals were not based on nutritional screening tool, but the subsequent clinical and nutritional situation. It is important that nutritional screening is correct so that there is a consistent referral process
Consideration of re-screening GO surgical patients day 5 post-surgery	− There is a high prevalence of acute nutritional complications and decline post-surgery − Leaving this to be picked up on weekly re-screening may delay identification and referral to dietitians, which could have clinical considerations including > five days minimal intake introducing potential for refeeding syndrome risk, the management of which could involve a longer hospital stay − Risk benefit analysis should be conducted to consider resource implications in terms of nursing time vs. potential overall savings in terms of reducing hospital admission length and reducing morbidity with more timely nutritional intervention
Explore different approaches to nutritional screening in this patient group	− Whether alternative approaches could better nutritionally assess this group should be considered. This would need careful consideration and agreement particularly if this would lead to a deviation from hospital standard processes, with the risk of variations in care − Employing simple, routinely available but more accurate measures of body composition could address this issue, such as CT. However, this data source is predominately confided to research setting with technology not immediately implementable in hospital settings
Increased funding for a specialist gynae-oncology surgical dietitian role with a research component should be considered	− Given the additional complexities and identified clinical and research needs, further dietetic funding could be warranted − Sufficient funding for a GO surgical specialist dietitian would provide the required capacity for training and the anticipated increase in referrals with more accurate nutritional screening − Increased dietetic input has the potential to improve nutritional and clinical outcomes. This could also result in cost savings but robust cost–benefit evaluation is required
Re-audit	− To assess the effectiveness of the above strategies and identify whether further improvements are required − This audit was predominantly collected by one dietitian, and therefore the results should be interpreted with due caution. Re-audit is essential and, where possible, two dietitians should simultaneously extract data and compare to ensure screening accuracy, referral consistency, and patient outcomes

## Data Availability

The data presented in this study are available from the corresponding author upon reasonable request. The data are not publicly available due to privacy and ethical restrictions.

## Appendix A

**Table A1:** Databases and search terms employed for literature search.

Database	Search Terms
PubMed	((“Nutritional Screening” OR “Nutritional Assessment” OR “nutritional screening”[tiab] OR “nutritional assessment”[tiab] OR “nutritional evaluation”[tiab] OR “malnutrition screening”[tiab] OR “nutrition risk”[tiab] OR “undernutrition screening”[tiab]) AND (“Surgical Procedures, Operative”[Mesh] OR “surgery”[tiab] OR “surgical”[tiab] OR “operative”[tiab] OR “surgical procedures”[tiab] OR “surgical patients”[tiab]) AND (“Gynecologic Neoplasms” OR “gynecologic oncology”[tiab] OR “gynaecologic oncology”[tiab] OR “gynecologic neoplasm*”[tiab] OR “gynaecologic neoplasm*”[tiab] OR “ovarian cancer”[tiab] OR “cervical cancer”[tiab] OR “endometrial cancer”[tiab] OR “uterine cancer”[tiab] OR “vulvar cancer”[tiab] OR “vaginal cancer”[tiab]))
Web of Science	(“nutritional screening” OR “nutritional assessment” OR “nutritional evaluation” OR “malnutrition screening” OR “nutrition risk” OR “undernutrition screening”) AND (“surgery” OR “surgical” OR “operative” OR “surgical procedures” OR “surgical patients”) AND (“gynecologic oncology” OR “gynaecologic oncology” OR “gynecologic neoplasm*” OR “gynaecologic neoplasm*” OR “ovarian cancer” OR “cervical cancer” OR “endometrial cancer” OR “uterine cancer” OR “vulvar cancer” OR “vaginal cancer”)
Medline via Ovid	1. exp Nutrition Screening.mp. 2. exp Nutritional Assessment/ 3. (“nutritional screening” OR “nutritional assessment” OR “nutritional evaluation” OR “malnutrition screening” OR “nutrition risk” OR “undernutrition screening”).[tiab]. 4. 1 OR 2 OR 3 5. exp Surgical Procedures, Operative/ 6. (surgery OR surgical OR operative OR “surgical procedures” OR “surgical patients”).[tiab]. 7. 5 OR 6 8. exp Gynecologic Neoplasms/ 9. (“gynecologic oncology” OR “gynaecologic oncology” OR “gynecologic neoplasm*” OR “gynaecologic neoplasm*” OR “ovarian cancer” OR “cervical cancer” OR “endometrial cancer” OR “uterine cancer” OR “vulvar cancer” OR “vaginal cancer”).[tiab].

**Table A2:** Eligibility criteria for inclusion in literature review.

Inclusion Criteria	Exclusion Criteria
Gynae-oncology surgical patients Assessment of nutrition in pre-, peri- or post-operative period Adults (18+ years old) English language Data where assessment of nutrition under routine clinical care can be extracted Published within the last 10 years to reflect improvements in clinical care and outcomes in this time, with advances in GO cancer diagnostics and surgical practices	Reviews Conference abstracts Not undergone or undergoing gynae-oncology (GO) surgery Non-English language <18 years old Mixed cohort where it is not possible to separate nutritional data for GO surgical patients Nutritional assessment integrated with other assessments (e.g., frailty), where results on nutrition status cannot be isolated Dietary/nutritional intervention studies where pre-intervention or without-intervention data cannot be isolated Focus on assessing nutritional status for another treatment modality, such as chemotherapy or radiotherapy, where nutritional data for surgery cannot be isolated Case studies Published >10 years ago No details on method of nutritional screening or assessment tool provided
